# Aetiology of pancytopenia: Experience of a South African tertiary academic centre

**DOI:** 10.4102/ajlm.v11i1.1645

**Published:** 2022-05-31

**Authors:** Erica-Mari Nell, Zivanai C. Chapanduka

**Affiliations:** 1 Division of Haematological Pathology, Department of Pathology, Faculty of Medicine and Health Sciences, Stellenbosch University, Cape Town, South Africa; 2 National Health Laboratory Service, Tygerberg Hospital, Cape Town, South Africa

**Keywords:** pancytopenia, sepsis, HIV, haematological malignancy, nutritional deficiency, megaloblastic anaemia, aging

## Abstract

**Background:**

Pancytopenia is a manifestation of numerous disease entities. The causes of pancytopenia differ with geographic region, socio-economic factors and HIV prevalence. Awareness of the common causes of pancytopenia may aid timely diagnosis.

**Objective:**

This study aimed to determine the aetiology of pancytopenia in a South African population.

**Methods:**

A retrospective observational study of adult patients presenting with pancytopenia at Tygerberg Academic Hospital, South Africa, from January 2016 to December 2017 was performed. Data on pancytopenia cases were obtained from the laboratory information system and utilised to determine the causes of pancytopenia.

**Results:**

A total of 673 cases of pancytopenia were identified. The most common causes of pancytopenia were chemoradiation therapy (25%), sepsis (18%), haematological malignancy (9%), advanced HIV (7%), and megaloblastic anaemia (6%). The diagnostic yield of bone marrow examinations (BME) was 57% (*n* = 52/91). The aetiology of pancytopenia differed according to age, with malignancy being a more common cause of pancytopenia among the elderly.

**Conclusion:**

Several easily recognisable and treatable conditions can manifest as pancytopenia. Prompt management of such conditions, notably sepsis and megaloblastic anaemia, can result in the resolution of the cytopenias and negate the need for a BME. However, haematological malignancy and unexplained pancytopenia strongly rely on a BME to establish a diagnosis. Pancytopenia investigations, when guided by appropriate clinic-laboratory findings, can promptly identify the underlying aetiology, while also identifying cases where an expedited BME is required. This is valuable in resource-conscious medicine.

## Introduction

The term pancytopenia is used to describe a reduction in all three haematopoietic cell lines in the peripheral blood, namely erythrocytes, leukocytes and platelets. Pancytopenia is not a disease entity, but rather the manifestation of several diseases and signifies the need for investigation. Chemotherapy and radiation therapy are predictable causes of pancytopenia.^1^ The occurrence of pancytopenia in the absence of chemotherapy or radiation therapy is a common diagnostic dilemma and a bone marrow examination (BME) is recommended if the cause of pancytopenia cannot be otherwise ascertained. Pancytopenia can also be caused by central inadequate production or peripheral destruction/sequestration,^1^ and the incidence of the various causes of pancytopenia differs based on geographic, socio-economic, dietary and other factors. In India, numerous studies have highlighted megaloblastic anaemia and aplastic anaemia as the commonly encountered causes of pancytopenia.^2,3,4,5,6,7,8,9,10,11,12,13,14,15^ Studies from neighbouring countries, including Pakistan,^16,17,18,19,20^ Bangladesh^21,22^ and Nepal,^23,24,25^ show similar findings. Climate also affects the aetiology of pancytopenia by affecting disease transmission.^26^ As such, malaria, which is endemic in Bangladesh, was found to be the most common cause of pancytopenia in a Bangladeshi study.^22^

In high-income countries, however, the disease profile is quite different. Studies done in France, Sweden, South Korea, Oman and the United States demonstrate that haematological malignancies (HMs) are the most common cause of pancytopenia.^27,28,29,30,31,32^ A study in Mexico revealed a mix of HM and megaloblastic anaemia as the most common causes,^33^ demonstrating the role that socio-economic status and access to healthcare has on the aetiology of pancytopenia.

In Africa, the causes of pancytopenia follow the pattern of poor nutrition and poor access to healthcare. Studies performed in Zimbabwe, Djibouti, Morocco and Tunisia showed that megaloblastic anaemia was the most common cause of pancytopenia.^34,35,36,37^ Furthermore, HIV was found to be the third most common cause of pancytopenia in both the Zimbabwean and Djiboutian studies.^34,35^

To our knowledge, a study by Retief and Heyns performed in Bloemfontein in 1976 is the only previous study investigating the aetiology of pancytopenia in South Africa.^38^ The most common cause of pancytopenia as identified in that study was bone marrow aplasia (49.0%), and over two-thirds of those cases were attributed to radiation or chemotherapy. Infectious agents (9.7%) were the second most common cause, followed by megaloblastic anaemia (9.6%).

The HIV pandemic started in 1981, thus the effect of HIV on pancytopenia was not seen in the Retief and Heyns study.^39^ There is currently a high burden of HIV infection in South Africa, and by the middle of 2019, 13.5% of South Africans, that is 7.97 million people, were living with HIV.^40^ Cytopenias are commonly observed in patients with HIV infection and are often multifactorial in aetiology.^41^ While cytopenias in HIV are common, the prevalence of pancytopenia in HIV clinics in African countries is low: 0.7% in an Ethiopian study and 0.5% in a Ugandan study.^42,43^ The pancytopenia prevalence is much higher (8.7%) in HIV clinics in Puerto Rico.^44^

This study aimed to contribute to the body of knowledge regarding the aetiology of pancytopenia in a developing country with a high burden of HIV and to assess the most common causes of pancytopenia across different age groups.

## Methods

### Ethical considerations

This study was approved by the Human Research Ethics Committee review board of Stellenbosch University (study approval number: S18/08/171) and all research was performed in accordance with relevant regulations with anonymised data. The requirement for informed consent was waived by the ethics committee due to the retrospective nature of the study.

### Setting, specimens and defining criteria

A retrospective cross-sectional descriptive study was conducted over a two-year period. All adult patients with new-onset pancytopenia who were treated at Tygerberg Academic Hospital, Cape Town, South Africa, from 01 January 2016 to 31 December 2017 were identified and included in our study.

Pancytopenia was defined as leucocyte count < 4 × 10^9^/L, haemoglobin < 10 g/dL, and a platelet count < 100 × 10^9^/L. These parameters are comparable with other studies investigating the aetiology of pancytopenia.^6,7,14,15,16,21,45,46^

Patients were excluded from the study if they received clinical care at another facility even though the BME was reported at Tygerberg Academic Hospital.

### Data collection and interpretation

The data were obtained from the laboratory information system (LIS) of the National Health Laboratory Service. An LIS search was conducted to identify cases meeting the study criteria for pancytopenia. The identified pancytopenia cases were reviewed on the National Health Laboratory Service LIS to identify the cause of the pancytopenia.

Information retrieved from the LIS included the reports of peripheral blood smears, BMEs, vitamin B12 and serum folate levels, iron studies, viral studies, *Mycobacterium tuberculosis* culture and GeneXpert^®^ tests (Cepheid, Sunnyvale, California, United States), malaria rapid tests (BinaxNOW Malaria, Abbott Laboratories, Chicago, Illinois, United States) and thick and thin smears, autoimmune screens, sepsis markers, and blood cultures. In addition, information on patient age, gender, HIV status, CD4 count, and HIV viral load was obtained. Advanced HIV was defined as a CD4 count < 200 cells/µL.^47,48^ Since the aetiology of cytopenias in HIV is multifactorial, evidence of contributing factors such as folate deficiency, opportunistic infection or malignancy were sought in HIV-positive patients. In the absence of any other cause of cytopenias, and if the CD4 count was < 200 cells/µL, the pancytopenia was attributed to the advanced HIV.

### Data analysis

Statistical analysis was done in conjunction with the Biostatistics Department of Stellenbosch University. Percentages were calculated for categorical variables. Age was presented as mean and standard deviation. Non-parametric data such as HIV viral load, CD4 count, time to BME and time to resolution of pancytopenia were presented as medians and interquartile ranges. Pearson’s chi-square tests were used to assess the statistical differences in the frequencies of causes of pancytopenia between the HIV-positive and negative groups, as well as between different age groups. The data were analysed using Statistical Package for the Social Sciences version 25 (IBM Corp., Armonk, New York, United States).

## Results

**FIGURE 1:**
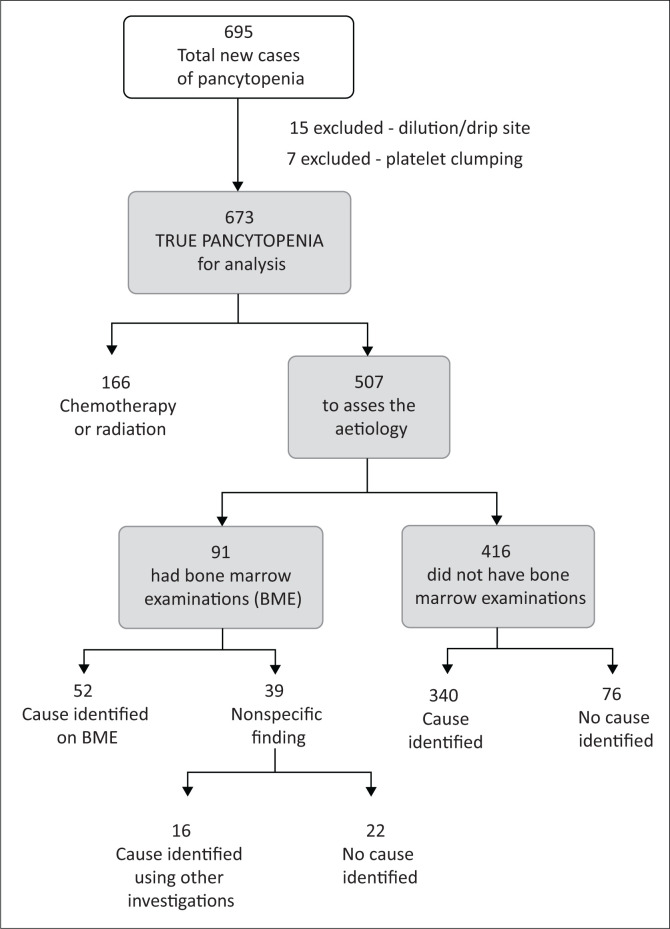
Flow diagram of case selection and stratification to identify the causes of pancytopenia at Tygerberg Academic Hospital, Cape Town, South Africa, from January 2016 to December 2017.

A total of 695 cases of new-onset pancytopenia were identified within the two-year period. Further investigation showed that seven cases had platelet clumping with a subsequent platelet count above 100 × 10^9^/L, and 15 cases were due to sample dilution, having been collected from a cannulated infusion vein. Thus the total number of true pancytopenia cases was 673 over the specified period (Figure 1).

True pancytopenia was confirmed in 673 cases. Chemoradiation therapy was found to be the cause in 166 cases, leaving 507 non-iatrogenic cases with unidentified underlying aetiologies. Of those, 91 patients had a BME and the cause of pancytopenia was identified in 52 of these patients. In 16 patients whose BME showed non-specific findings, a cause for pancytopenia was found using information available on the LIS. Using information available on the LIS, the cause of pancytopenia was identified in 340 of the remaining 416 patients who did not have a BME.

Of the 673 patients, 273 (41%) were male and 400 (59%) were female. The mean age at which pancytopenia was diagnosed was 44 ± 15 years (range: 18–87 years).

### Most common causes of pancytopenia

**FIGURE 2:**
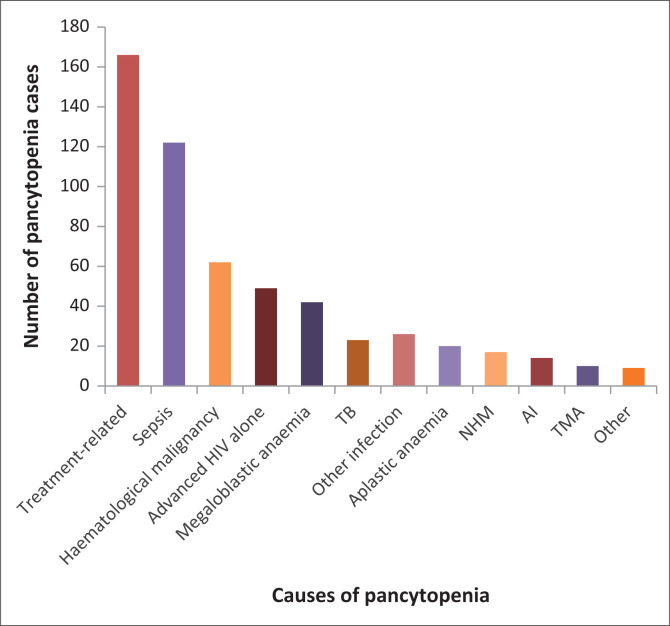
Frequency of different causes of pancytopenia among adult patients at Tygerberg Academic Hospital, Cape Town, South Africa, from January 2016 to December 2017. Note: Top five causes: Chemoradiation therapy (25%); Sepsis (18%); Haematological malignancy (9%); Advanced HIV (7%); Megaloblastic anaemia (6%). *Other infections* include malaria, cytomegalovirus, parvovirus B19, Epstein-Barr virus and hepatitis B virus. AI, autoimmune; NHM, non-haematological malignancy; TB, tuberculosis; TMA, thrombotic microangiopathy.

**FIGURE 3:**
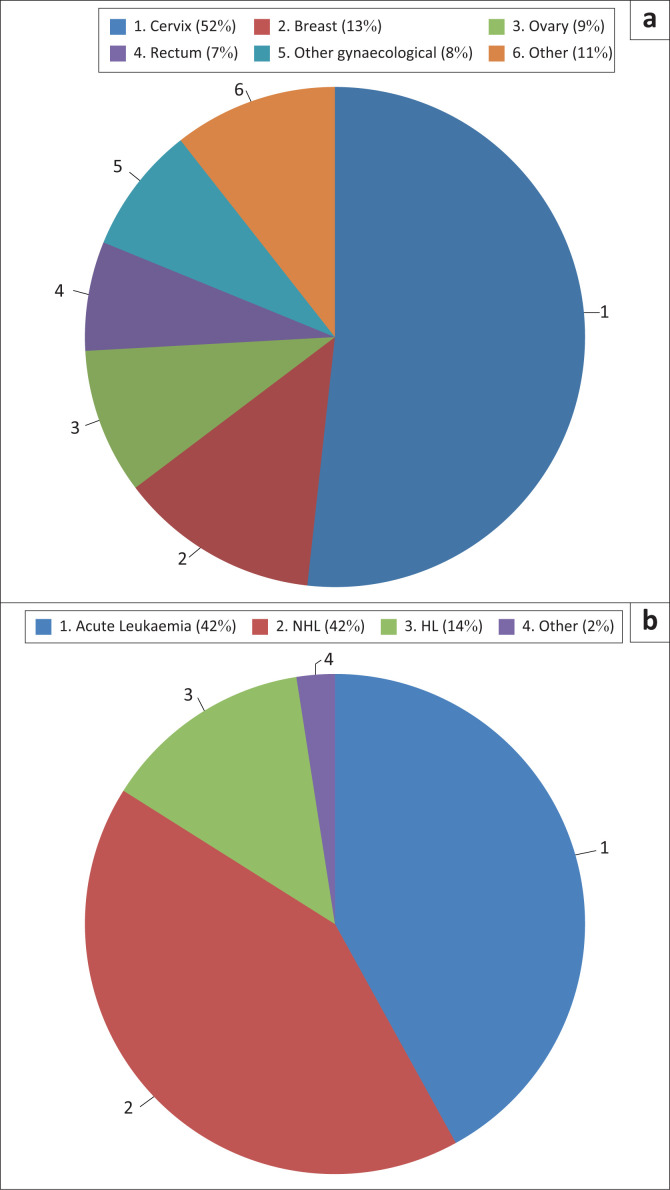
Pancytopenia cases at Tygerberg Academic Hospital, Cape Town, South Africa, from January 2016 to December 2017 for which chemoradiation therapy was the cause. (a) Site of NHM, (b) Haematological malignancy (HM) subtypes. Note: *Other gynaecological malignancies* include endometrial-, vulval- and chorio-carcinoma. HL, Hodgkin lymphoma; HM, haematological malignancy; NHL, non-Hodgkin lymphoma; NHM, non-haematological malignancy.

Chemotherapy and/or radiation therapy was the most common (25%; 166/673) cause of pancytopenia (Figure 2). The chemoradiation therapy was for the treatment of non-haematological malignancies (51%; 85/166) and HM (49%; 81/166) (Figure 3).

The most common causes of pancytopenia among the remaining 507 (75%) cases were sepsis (18%; 122/673), HM (9%; 62/673), advanced HIV with no other identifiable cause (7%; 49/673) and megaloblastic anaemia (6%; 42/673). A cause for pancytopenia could not be established in 15% (98/673) of cases.

Haematological conditions were found to be common causes of pancytopenia. These included HM (9%; 62/673) and aplastic anaemia (3%; 20/673). Six of the 20 patients with aplastic anaemia had a paroxysmal nocturnal haematuria clone. Serum vitamin B12, folate and ferritin levels were measured in 280 of the 507 patients with non-iatrogenic pancytopenia. Of these, 12% (33/280) had isolated folate deficiency and 3% (9/280) had vitamin B12 deficiency.

Infection contributed to a considerable proportion of pancytopenia cases. Sepsis was the most common non-iatrogenic cause of pancytopenia. In 80% of sepsis cases (97/122), a blood culture was positive for an organism. Gram-negative bacteria were more commonly cultured (61%; 74/122) compared to Gram-positive bacteria (16%; 19/122). The remaining four cultures were positive for *Candida albicans*. In 62% (76/122) of sepsis-associated pancytopenia cases, the pancytopenia resolved. The median time to resolution of pancytopenia was 2 days (interquartile range: 1–6). Twenty-three (3%; 23/673) patients had positive *Mycobacterium tuberculosis* cultures/GeneXpert^®^; the majority (19/23) of these were HIV-positive patients. Nine (1%; 9/673) patients had malaria.

### Description of pancytopenia in patients with HIV

Of the 507 non-iatrogenic pancytopenia cases, 41% (207/507) were HIV-positive, 47% (236/507) were HIV-negative, and the HIV status was unknown in 13% (64/507). The median CD4 count was 94 cells/µL (interquartile range: 33–202 cells/µL, *n* = 175 patients tested) and the median HIV viral load was 1096 copies/mL (interquartile range lower than detectable limit – 91 560 copies/mL, *n* = 113 patients tested). The viral load was undetectable in 14% (29/207) of the HIV-positive patients with pancytopenia, and advanced HIV was seen in 68% (141/207). The CD4 count was not performed in 27 HIV-positive patients with pancytopenia, thus the proportion of patients with advanced HIV may be underestimated. Further investigation in patients with advanced HIV revealed additional contributing causes of pancytopenia, including sepsis (22%; 31/141), folate deficiency (13%; 18/141) and tuberculosis (10%; 14/141). Moreover, advanced HIV was the only identifiable cause of pancytopenia in 24% (49/207) of the HIV-positive patients, accounting for 7% of all cases (49/673).

**FIGURE 4:**
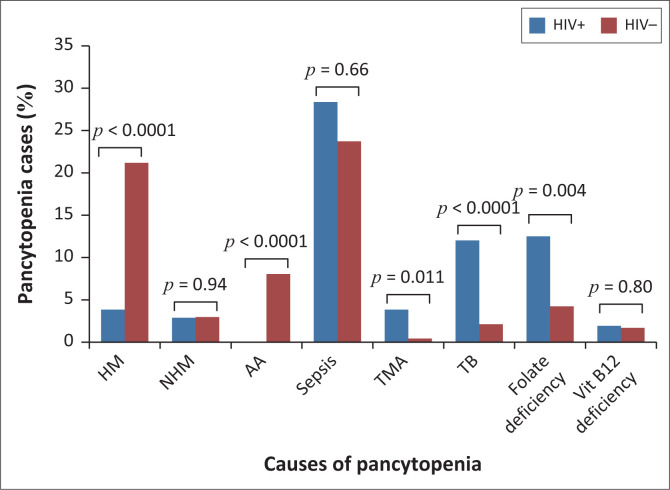
Proportions of various causes of pancytopenia among HIV-positive and HIV-negative patients at Tygerberg Academic Hospital, Cape Town, South Africa, from January 2016 to December 2017. HM, haematological malignancy; NHM, non-haematological malignancy; TMA, thrombotic microangiopathy; TB, tuberculosis; Vit, vitamin; AA, aplastic anaemia.

The aetiology of pancytopenia differed between HIV-positive and HIV-negative patients (Figure 4). Haematological malignancies and aplastic anaemia were significantly more common in HIV-negative patients (*p* < 0.0001), while folate deficiency (*p* = 0.004), tuberculosis (*p* < 0.0001) and thrombotic microangiopathy (*p* = 0.011) were more common among HIV-positive patients. There was no significant difference in the proportion of sepsis cases between the HIV-positive and HIV-negative groups (*p* = 0.66).

### Bone marrow examination to determine pancytopenia aetiology

Bone marrow examination was performed to determine the cause of pancytopenia in 91 of the non-iatrogenic cases and this revealed the cause of pancytopenia in 52 cases (57%; 52/91). Thirty-six of these had HM (40%; 36/91), nine had aplastic anaemia (10%; 9/91), and three had non-haematological malignancies (3%; 3/91). The most common HMs were myelodysplastic syndrome (11%) and acute leukaemia (10%). Notably, in four of the nine acute leukaemia cases, there were scanty or no blasts (< 1%) on the peripheral blood smear. In the remaining 39 BMEs, no specific bone marrow pathology could be identified.

### Causes of pancytopenia in different age groups

**FIGURE 5:**
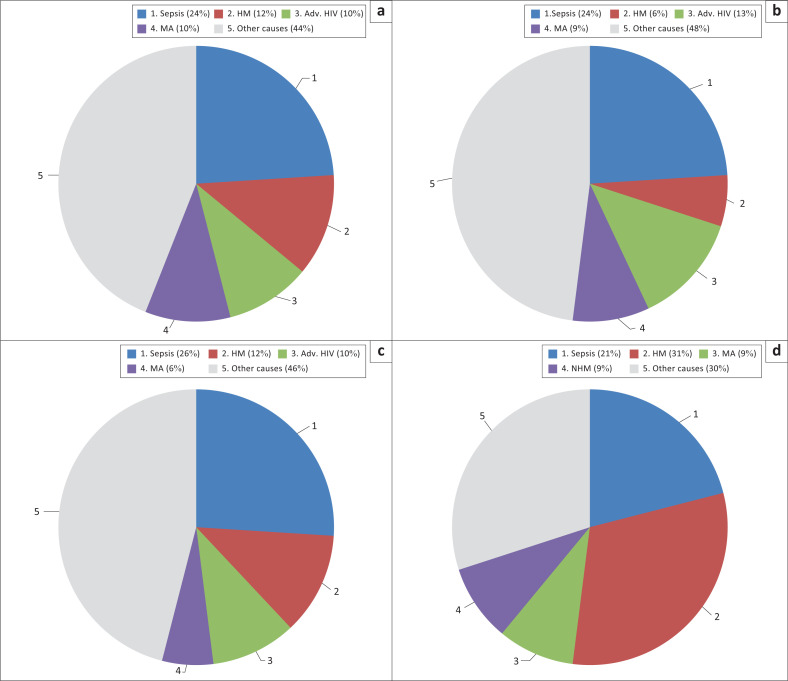
Most common causes of non-treatment-related pancytopenia among adult patients at Tygerberg Academic Hospital, Cape Town, South Africa, from January 2016 to December 2017. (a) All patients (*N* = 507), (b) 18–39 years old (*n* = 250), (c) 40–59 years old (*n* = 172), (d) 60–89 years old (*n* = 85). Adv. HIV, advanced HIV; HM, haematological malignancy; MA, megaloblastic anaemia; NHM, non-haematological malignancy.

The aetiology of pancytopenia also varied according to age (Figure 5). While sepsis was the most common cause of pancytopenia among young (18–39 years old) and middle-aged (40–59 years old) patients, HM was the most common cause of pancytopenia in the oldest age group (60–89 years old). There was a significantly higher proportion of patients diagnosed with HM (*p* < 0.0001) and non-haematological malignancies (*p* = 0.012) among patients aged 60–89 years old. The increase in HM seen in the 60–89-year-old category was mainly due to an increase in the number of myelodysplastic syndrome cases. The number of cases of sepsis, megaloblastic anaemia and aplastic anaemia did not significantly differ between the age categories.

## Discussion

This study showed that chemoradiation therapy was the most common cause of pancytopenia in adult patients at a South African tertiary academic centre. The most common non-treatment-related causes of pancytopenia were sepsis, HM, advanced HIV, and megaloblastic anaemia.

The aetiology of pancytopenia as found in our study is comparable to the findings of a previous South African tertiary institution study.^38^ Their study showed chemoradiation therapy to be the most common cause of pancytopenia and revealed infection as another prominent cause.^38^ Haematological malignancy, which was not a prominent cause of pancytopenia in their study, was the third most common cause of pancytopenia in this study.

Infection, megaloblastic anaemia and aplastic anaemia are common causes of pancytopenia in developing countries while HM is the most common cause in developed countries (Table 1). In comparison to the aetiologies of pancytopenia described by studies across the world, our study shows features common to both developing and developed countries. This is similar to the findings of a study performed in Mexico where the most common causes of pancytopenia were HM and megaloblastic anaemia.^32^ In South Africa, there are considerable differences in access to health services between socio-economic groups, and the poor face many predisposing factors that are recognised as social determinants of ill health.^49^ HIV and other communicable diseases are concentrated among the lower socio-economic groups of South Africa.^50^ This may explain why infections contributed considerably to the aetiology of pancytopenia in our study.

**TABLE 1:** Most common causes of pancytopenia across different studies globally, 1976–2019.

Continent	Study	Country	*N*	Most common cause (%)	Second most common cause (%)	Third most common cause (%)
Africa	This study	South Africa	673	Treatment (25.0)	Sepsis (18.0)	Haematological malignancy (9.0)
	Retief & Heyns^38^	South Africa	132	Hypoplastic anaemia (49.0)	Infection (9.7)	Megaloblastic anaemia (9.6)
	Savage et al.^34^	Zimbabwe	134	Megaloblastic anaemia	Aplastic anaemia	AIDS (-)
	Lavigne et al.^35^	Djibouti	81	Megaloblastic anaemia (49.0)	Hypersplenism (9.0)	HIV (6.0)
	Nafil et al.^36^	Morocco	118	Megaloblastic anaemia (32.0)	Acute leukaemia (24.0)	Aplastic anaemia (15.0)
	Klii et al.^37^	Tunisia	103	Megaloblastic anaemia (36.0)	Infections (15.0)	Haematological malignancy (13.0)
Asia	Jain et al.^12^	India	250	Hypersplenism (29.0)	Infection (26.0)	Treatment (17.0)
	Dasgupta et al.^13^	India	248	Aplastic anaemia (33.0)	Megaloblastic anaemia (21.0)	Leishmaniasis/ Hypersplenism (14.0)
	Basavaiah et al.^22^	Bangladesh	400	Malaria (27.0)	Megaloblastic anaemia (23.0)	Chronic liver disease (14.0)
	Biswas et al.^21^	Bangladesh	80	Megaloblastic anaemia (35.0)	Aplastic anaemia (33.0)	Acute leukaemia (8.0)
	Tareen et al.^54^	Pakistan	180	Malaria (29.0)	Tuberculosis (17.2)	Acute leukaemia (16.6)
	Rehmani et al.^20^	Pakistan	244	Aplastic anaemia (27.0)	Megaloblastic anaemia (20.0)	Acute leukaemia (14.0)
	Jha et al.^23^	Nepal	148	Hypoplastic anaemia (29.0)	Megaloblastic anaemia (24.0)	Haematological malignancy (22.0)
	Pathak et al.^25^	Nepal	102	Hypoplastic anaemia (42.0)	Megaloblastic anaemia (12.0)	Acute leukaemia (9.0)
	Hamid & Shukry^55^	Yemen	75	Hypersplenism (28.0)	Malaria (17.0)	Megaloblastic anaemia (15.0)
	Al-Khalisi et al.^30^	Oman	105	Acute leukaemia (30.0)	Aplastic anaemia (17.0)	Megaloblastic anaemia (13.0)
	Yokus & Gedik^45^	Turkey	137	Megaloblastic anaemia (17.0)	Chronic liver disease (15.0)	Malignancy (13.0)
	Jalaeikhoo et al.^46^	Iran	665	Acute leukaemia (35.0)	MDS (15.0)	Megaloblastic anaemia (14.0)
	Bae et al.^29^	South Korea	901	MDS (17.0)	AML (15.0)	Lymphoma (9.0)
	Azaad et al.^56^	China	25	Megaloblastic anaemia (28.0)	Aplastic anaemia (20.0)	MDS (12.0)
Europe	Imbert et al.^27^	France	213	Myeloid malignancy (42.0)	Aplastic anaemia (10.0)	Megaloblastic anaemia (8.0)
	Keisu and Ost.^28^	Sweden	100	Treatment (32.0)	Hypoplastic anaemia (19.0)	-
North America	Weinzierl & Arber.^31^	United States	250	MDS (28.0)	Acute leukaemia (24.0)	Aplastic anaemia (8.0)
	Devitt et al.^32^	United States	132	Haematological malignancy (64.0)	Aplastic anaemia (4.0)	Megaloblastic anaemia (2.0) and HIV (2.0)
	Vargas-Carretero et al.^33^	Mexico	109	MDS (20.0)	Megaloblastic anaemia (18.0)	AML (12.0)

AML, acute myeloid leukaemia; MDS, myelodysplastic syndrome.

Advanced HIV was found to be one of the common causes of pancytopenia in our study, similar to studies performed in Zimbabwe, Djibouti, India and the United States.^12,32,34,35^ In our study, 41% of patients were HIV-positive. The majority of these patients had advanced HIV, and in a quarter of these, advanced HIV was found to be the only identifiable cause of pancytopenia. Additionally, our study showed that HIV status alters the aetiology of pancytopenia; folate deficiency, tuberculosis and thrombotic microangiopathy were significantly more common among HIV-positive patients, while aplastic anaemia and HM were significantly more common among HIV-negative patients. Interestingly, despite the known risk of HIV-associated lymphomas in advanced HIV,^51^ we did not find an increase in HM-associated pancytopenia in HIV-positive patients. This may be because lymphomas do not typically cause pancytopenia even when there is bone marrow infiltration.^52^

Haematological malignancies were a more common cause of pancytopenia in our study than in other studies performed in developing nations (Table 1). The burden of cancer is highest in affluent regions and is thought to be associated with diet, lifestyle and environmental exposures. With increasing urbanisation and globalisation, developing nations are increasingly exposed to diet and lifestyle changes, including smoking, a sedentary lifestyle and obesity, which were previously mainly seen in developed societies.^53^ In addition, developing nations have an increased risk of viral infection, including infection with oncoviruses, which can lead to malignancy.^53^ Expensive diagnostic approaches are also increasingly available in tertiary centres, allowing for easier diagnosis of malignancy, which may be missed in rural regions or countries with fewer resources. These factors are likely contributing to the changing landscape of pancytopenia aetiology in South Africa.

Megaloblastic anaemia as a cause of pancytopenia was not as prevalent in our study as in studies performed in India^3,4,5,7,8,9,10,11,13,15^ and the rest of Africa.^34,35,36,37^ This is likely because the South African government introduced legislation in 2003 for the mandatory folate fortification of the staple maize meal and wheat flour.^57,58^ Nevertheless, folate deficiency was more common in HIV-positive patients than in HIV-negative patients in this study, likely reflecting poor folate absorption due to HIV-associated gastrointestinal disease. Importantly, six of the 48 patients with megaloblastic anaemia in our study had an underlying pathology leading to depletion of folate reserve. This highlights the importance of follow-up in patients with haematinic deficiencies and the importance of further investigation if there is a poor response to the haematinic replacement or clinical suspicion of an underlying condition.

The aetiology of pancytopenia also varied with age. There were significantly more malignancies, both haematological and non-haematological, in the age group 60–89 years. This is comparable to the findings of studies in Turkey and Iran.^45,46^ Globally, malignancies are not a more common cause of pancytopenia in older patients. In studies conducted in India, megaloblastic anaemia was found to be the most common cause of pancytopenia, with no noticeably higher frequency of malignancy among older people.^6,12,15^ The substantial variation in the frequency of malignancy as a cause of pancytopenia in the elderly is most likely due to regional risk factors for megaloblastic anaemia, as well as variable availability of expensive diagnostic approaches such as flow cytometry, cytogenetics and next-generation sequencing for the diagnosis of malignancy.

The findings of this study highlight that good clinical and laboratory correlation is required for prompt and cost-effective identification of the underlying cause of pancytopenia to guide management. Chemoradiation is a predictable cause of pancytopenia; however, cell counts should also be monitored, and if the cell counts do not improve, investigation for vitamin B12/folate deficiency or marrow infiltration should be performed. Sepsis was another easily identifiable cause of pancytopenia in our study. The median time to resolution of sepsis-induced pancytopenia was two days. Therefore, when sepsis is treated empirically, cell counts should be monitored for recovery. Should cell counts fail to recover, further investigation is required. Vitamin B12 and folate deficiency are important to exclude in cases of pancytopenia. In cases where haematinic deficiency is confirmed based on serum levels, poor response to replacement therapy may indicate underlying marrow pathology requiring BME. Knowledge of the vitamin B12 and folate serum levels are also important for the interpretation of BMEs, especially if myelodysplastic syndrome is suspected. Additionally, if there are features of myelodysplastic syndrome, acute leukaemia or bone marrow infiltration (such as unexplained leucoerythroblastic reaction) in peripheral blood or, importantly, if the pancytopenia is unexplained, a BME should be fast-tracked in the investigation of the pancytopenia. Furthermore, in patients with HIV, vitamin B12 and folate deficiency should be excluded early, as folate deficiency was found to be common in the HIV patients in our study. Moreover, in HIV-positive patients, there should be a thorough investigation for tuberculosis, and a BME is recommended to investigate marrow infiltration by opportunistic infections and malignancy. In older patients, there should be a high clinical suspicion of malignancy. Ultimately, BME remains a valuable procedure for the investigation of unexplained pancytopenia.

### Limitations

One of the limitations of our study was that only retrospective information available on the LIS was used to identify the cause of pancytopenia. As a result, the contribution of drugs and co-morbidities was unknown and a cause for pancytopenia could not be identified in 14% of patients. The World Health Organization defines advanced HIV as either CD4 count < 200/µL or clinical stage III or stage IV disease. However, the clinical stage of HIV disease was not known in this study. Thus the definition of advanced HIV was based only on CD4 count, and the patients with advanced HIV may be underestimated.^48^ Clinicopathological correlation would also have improved the study. Furthermore, it is noteworthy that our study was performed in a tertiary hospital, thus selection bias may have resulted in chemoradiation therapy being the most common cause of pancytopenia.

Socio-economic, geographical and ethnic factors are known to influence the aetiology of pancytopenia. It is noteworthy, however, that differences in study design also influence the described aetiology of pancytopenia reported by different studies. Study designs differ in their definitions of pancytopenia and whether clinical or laboratory data or both were used. Additionally, some studies only evaluate BME patients, while some include all patients with pancytopenia. Some studies may also include children, and others may include patients that had chemotherapy or radiation therapy. These differences influence the top causes of pancytopenia between different studies.

### Conclusion

This study shows that the most common causes of new-onset pancytopenia in adults at a South African tertiary academic centre are chemoradiation therapy, sepsis, HM, advanced HIV and megaloblastic anaemia. These results demonstrate the need for the prompt recognition and treatment of sepsis and megaloblastic anaemia, early recognition of HIV and initiation of antiretroviral therapy, and a thorough investigation for malignancy. Integration of clinical, laboratory and radiological findings to guide investigation of pancytopenia allows for prompt diagnosis, while also elucidating where BME should be expedited in the investigation of pancytopenia.
